# Method comparison for the direct enumeration of bacterial species using a chemostat model of the human colon

**DOI:** 10.1186/s12866-019-1669-2

**Published:** 2020-01-02

**Authors:** Ines B. Moura, Charmaine Normington, Duncan Ewin, Emma Clark, Mark H. Wilcox, Anthony M. Buckley, Caroline H. Chilton

**Affiliations:** 1 Leeds Institute of Medical Research, Faculty of Medicine and Health, University of Leeds, Leeds, UK; 2 Department of Microbiology, Leeds Teaching Hospitals NHS Trust, Leeds, UK

**Keywords:** Bacterial culture, Real-time quantitative PCR, *C. difficile*, FMT, Chemostat gut model

## Abstract

**Background:**

*Clostridioides difficile* infection (CDI) has a high recurrent infection rate. Faecal microbiota transplantation (FMT) has been used successfully to treat recurrent CDI, but much remains unknown about the human gut microbiota response to replacement therapies. In this study, antibiotic-mediated dysbiosis of gut microbiota and bacterial growth dynamics were investigated by two quantitative methods: real-time quantitative PCR (qPCR) and direct culture enumeration, in triple-stage chemostat models of the human colon. Three *in vitro* models were exposed to clindamycin to induce simulated CDI. All models were treated with vancomycin, and two received an FMT. Populations of total bacteria, *Bacteroides* spp., *Lactobacillus* spp., *Enterococcus* spp., *Bifidobacterium* spp., *C. difficile,* and Enterobacteriaceae were monitored using both methods. Total clostridia were monitored by selective culture. Using qPCR analysis, we additionally monitored populations of *Prevotella* spp., *Clostridium coccoides* group, and *Clostridium leptum* group.

**Results:**

Both methods showed an exacerbation of disruption of the colonic microbiota following vancomycin (and earlier clindamycin) exposure, and a quicker recovery (within 4 days) of the bacterial populations in the models that received the FMT. *C. difficile* proliferation, consistent with CDI, was also observed by both qPCR and culture. Pearson correlation coefficient showed an association between results varying from 98% for *Bacteroides* spp., to 62% for Enterobacteriaceae.

**Conclusions:**

Generally, a good correlation was observed between qPCR and bacterial culture. Overall, the molecular assays offer results in real-time, important for treatment efficacy, and allow the monitoring of additional microbiota groups. However, individual quantification of some genera (e.g. clostridia) might not be possible without selective culture.

## Background

*Clostridioides difficile* infection (CDI) is a healthcare-associated infection related with high morbidity, mortality and costs [1]. Vancomycin and fidaxomicin are the recommended antibiotics for the treatment of moderate to severe CDI, but recurrent infection rates can be as high as 20–30% [1–4]. Faecal microbiota transplantation (FMT) has been used successfully as treatment for recurrent CDI (rCDI), with reported resolution rates that can reach ~ 90% [5–7]. However, much remains unknown about the human gut microbiota composition and behaviour, particularly in undefined microbiota replacement therapy, such as FMT.

The chemostat model has been validated against the intestinal contents of sudden-death victims and consists on a reliable representation on the microbial content and bacterial activities of the human colon [8]. This model of the human colon has been used to investigate the propensity of different antibiotics to induce CDI and the efficacy of treatments [9–15]. The results observed in the gut model have shown to correlate well with phase three clinical trials [9, 14], underlining the clinical relevance of this system. *Bacteroides* spp., *Bifidobacterium* spp. and *Clostridium* spp., are examples of bacterial genera highly abundant in the human gut that are often disrupted by antimicrobial therapy, as shown by *ex vivo* and *in vitro* studies [5, 12–14, 16–20]. The decline of microbial populations creates a potential niche for *C. difficile* spore germination, cell proliferation and toxin production, leading to CDI [9]. In the previous gut model studies, variations in microbial populations were investigated using bacterial culture. The accuracy of the gut model studies predicting clinical outcomes suggests that the bacterial populations targeted provide a suitable representation of the healthy human microbiota [17]. However, some important groups of the human gut microbiota are difficult to monitor by this method due to taxonomic complexity. Furthermore, bacterial culture is demanding on staff time and requires between 24 h and 48 h of plate incubation for colony growth, which can delay interventions to the model; e.g. commencement of antibiotic course during simulated CDI. Similar to bacterial culture, real-time PCR methodology can be used to investigate gut microbiota variations from a quantitate perspective [19–23], but populations targeted by this method may differ from those investigated by direct culture. Taxonomic profiling using 16S sequencing can also be used to increase our understanding of the gut microbiota populations exposed to antibiotic pressure [5, 18, 24]. However, the variations observed by 16S sequencing are qualitative, as bacterial groups differ in the number of 16S gene copies encoded on their genomes. Additionally, gut microbiota exposure to antibiotics can create ‘artefacts’, as the proportion of reads assigned to a bacterial population may increase as result of the depletion of abundant bacterial populations, and not due to bacterial expansion.

To better understand the dynamics of bacterial populations during replacement therapy and infection, we investigated the gut microbiota reconstitution following a simulated FMT, alongside an experiment of rCDI, using *in vitro* chemostat models of the human colon. Direct enumeration of key bacterial groups was monitored using two quantitative methodologies: selective bacteriological agars, and real-time quantitative PCR (qPCR). The advantages and disadvantages of each methodology, when applied to studies of the human gut microbiota, is investigated.

## Results

### Bacterial populations monitored by qPCR and bacterial culture

**Fig. 1:**
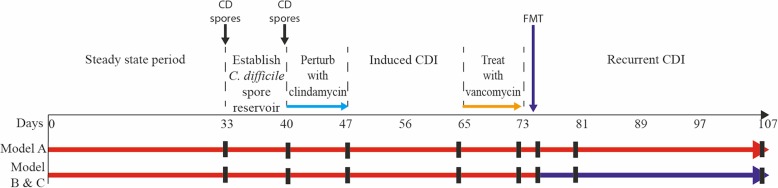
Experimental timeline for the recurrent CDI model A, and the faecal microbiota transplantation (FMT) models B and C. All models followed the same experimental design to induce and treat simulated CDI with clindamycin and vancomycin, respectively. Following treatment course with vancomycin, model A received no further intervention (red arrow), whereas model B and C received an FMT three days post vancomycin treatment (purple arrow). Black lines indicate the times at which samples were collected for qPCR and culture analysis

Gut microbiota variations were monitored in three chemostat models of the human colon, here described as A, B and C. Project outline is shown in Fig. 1. All models were started with the same human faecal slurry and underwent clindamycin induced CDI, followed by treatment with vancomycin. Up to the end of vancomycin treatment all models experienced the same experimental design, representing technical replicates. Bacterial populations were monitored in vessel 3 of model A, B and C throughout the experiment, using bacterial culture and qPCR assays. As the bacterial populations showed minor variations, only the mean logarithm values of the three models is shown. Following vancomycin instillation, model A was left without additional treatment, representing simulated rCDI. Three days post completion of vancomycin treatment, models B and C received FMT therapy. This three-day period allowed the washout of vancomycin to prevent residual antibiotic effects on the transplanted microbiota. The post-FMT results are shown as the mean of model B and C data, or model A only.

Real-time PCR assays species- and group-specific were applied to DNA extracted from gut model samples at key stages of the experiment to monitor population dynamics. Furthermore, total bacteria, *Bacteroides* spp., *Lactobacillus* spp., *Enterococcus* spp., *Bifidobacterium* spp., and *C. difficile* were also monitored using culture assay.

**Fig. 2:**
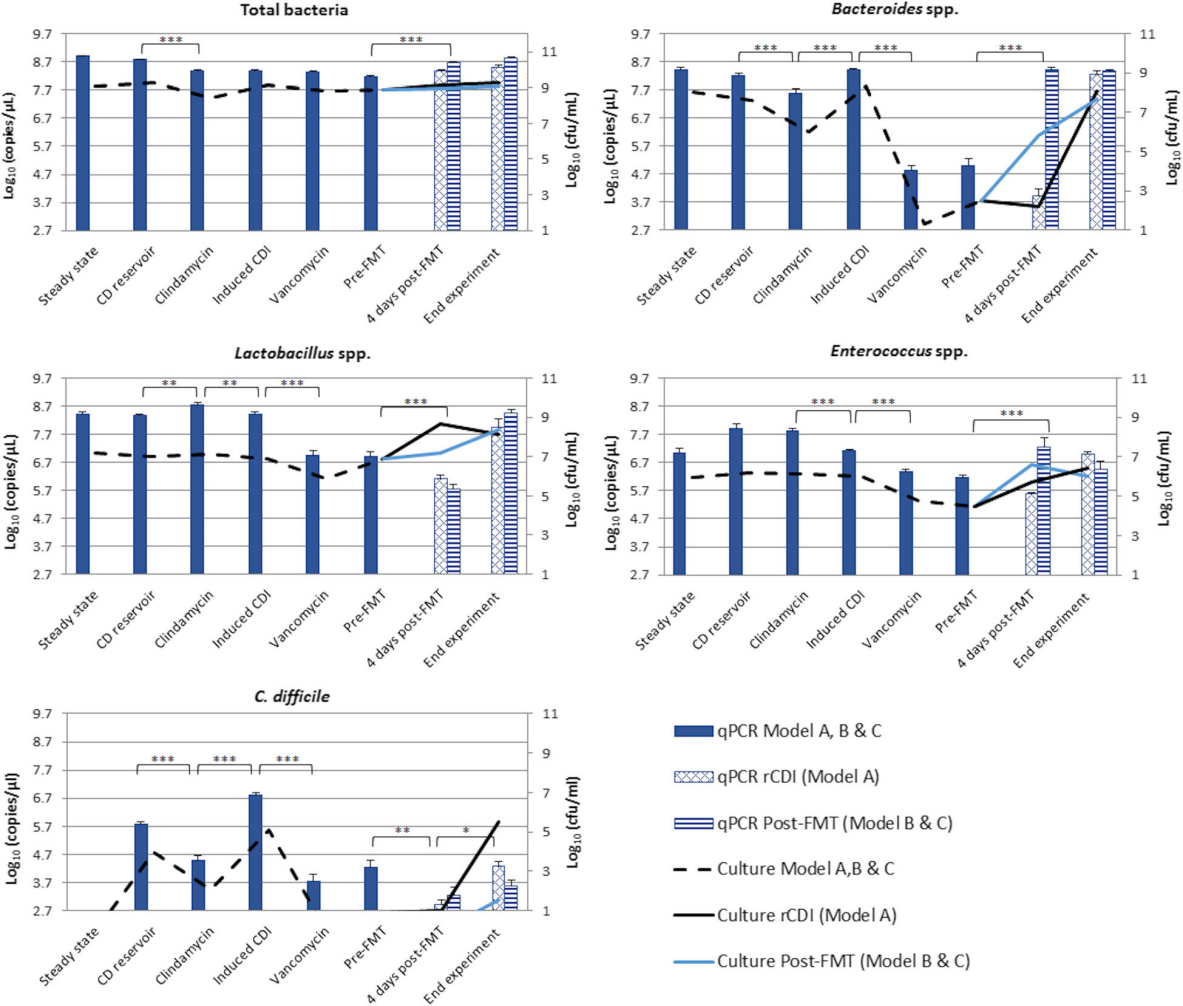
Mean gut microbiota populations of (**a**) total bacteria, (**b**) *Bacteroides* spp., (**c**) *Lactobacillus* spp., (**d**) *Enterococcus* spp., and (**e**) *C. difficile* in vessel 3 of model A, B and C at the different stages of the experiment. Bars represent the levels in log_10_ copies/μL measured by qPCR, and lines represent the populations levels in log_10_ cfu/mL measured by bacterial culture. CD, *C. difficile*; rCDI, recurrent CDI; FMT, faecal microbiota transplantation. Asterisks represent significant variations by qPCR between time points: *correspond to *p* < 0.05, **correspond to *p* < 0.005, and ***correspond to *p* < 0.0005

qPCR and bacterial culture analyses showed that the total bacterial populations remained stable throughout the experiment except for a decline of ~ 1 log_10_ copies/μL and ~ 1 log_10_ cfu/mL, respectively, observed following clindamycin dosing. The qPCR data showed this decline to be significant (*p* < 0.005) (Fig. 2a). qPCR data also showed a significant (*p* < 0.005) increase in total bacteria following gut microbiota reconstitution by FMT in models B and C. For the *Bacteroides* spp. population (Fig. 2b), clindamycin instillation caused a significant decline of ~ 1 log_10_ copies/μL (*p* < 0.0005), followed by recovery (*p* < 0.005) during CDI phase. Vancomycin dosing led to a ~ 4 log_10_ copies/μL decline (*p* < 0.005). In model A, *Bacteroides* spp. recovered only by the end of the experiment, whereas in model B and C an increase of ~ 3.5 log_10_ copies/μL (*p* < 0.0005) was observed four days after FMT. Bacterial culture results showed similar variations in *Bacteroides* spp., namely a ~ 2 log_10_ cfu/mL decline was observed following clindamycin dosing and ~ 7 log_10_ cfu/mL decline was observed following vancomycin instillation, with a faster recovery to steady state levels being observed in model B and C. Analysis by qPCR showed significant declines in *Lactobacillus* spp. (*p* < 0.005) (Fig. 2c), and *Enterococcus* spp. (*p* < 0.0005) (Fig. 2d) populations during CDI period, followed by a further decline caused by vancomycin instillation (*p* < 0.0005). Culture populations of *Lactobacillus* spp. and *Enterococcus* spp. remained stable up to vancomycin dosing that caused a ~ 1.0 log_10_ cfu/mL decline in both populations. In model B and C, both qPCR and culture data showed a recovery of *Enterococcus* spp. populations to steady state levels following FMT.

*Bifidobacterium* spp. populations analysed by qPCR were stable prior to clindamycin instillation, which caused a ~ 1 log_10_ copies/μL (*p* < 0.005) decline to ~ 7 log_10_ copies/μL, followed by recovery to steady state levels during CDI (*p* < 0.005). Vancomycin instillation also caused a decline of ~ 1.5 log_10_ copies/μL (*p* < 0.0005). No significant variations were observed in model B and C following FMT. In all models, *Bifidobacterium* spp. populations recovered by the end of the experiment. Culture-based analysis showed a depletion of *Bifidobacterium* spp. during clindamycin (~ 6 log_10_ cfu/mL) and vancomycin to below the culture assay limit of detection. In model A, *Bifidobacterium* spp. remained undetected by culture until the end of the experiment. Following FMT, *Bifidobacterium* spp. increased ~ 2.5 log_10_ cfu/mL in model B and C and recovered to steady state levels (~ 8 log_10_ cfu/mL) by the end of the experiment.

*C. difficile* was investigated using qPCR assays for the 16S gene and the housekeeping gene *gluD* (Fig. 2e and Additional file 1: Figure S1). In both assays, *C. difficile* copy number declined ~ 1.5 log_10_ copies/μL (*p* < 0.0005) during clindamycin instillation. This was followed by ~ 2.2 log_10_ copies/μL increase (*p* < 0.0005) post antibiotic period, consistent with *C. difficile* cell proliferation in the gut models, and was accompanied by toxin production, corresponding to simulated CDI. Average toxin value prior to vancomycin treatment was 2 relative units (RUs). Vancomycin treatment caused a decline in *C. difficile* populations of ~ 3 log_10_ copies/μL (*p* < 0.0005) and toxin declined to undetectable levels. In model A, a ~ 1.4 log_10_ copies/μL increase in *C. difficile* was observed towards the end of the experiment (*p* < 0.05). This was accompanied by toxin production corresponding to 3 RUs, which is consistent with rCDI. Following the FMT in models B and C, no significant increase in *C. difficile* copy number was observed and no toxin was detected, suggesting resolution of CDI (Fig. 2e). Monitoring of *C. difficile* by culture showed a *~* 2 log_10_ cfu/mL decline in *C. difficile* counts following clindamycin dosing. *C. difficile* population then increased *~* 3 log_10_ cfu/mL consistent with cell proliferation. *C. difficile* declined to below the limit of detection with vancomycin instillation and remained low up to the end of the experiment when a sudden increase consistent with spore germination and cell proliferation, was observed in model A only.

### Gut microbiota populations monitored by qPCR

**Fig. 3:**
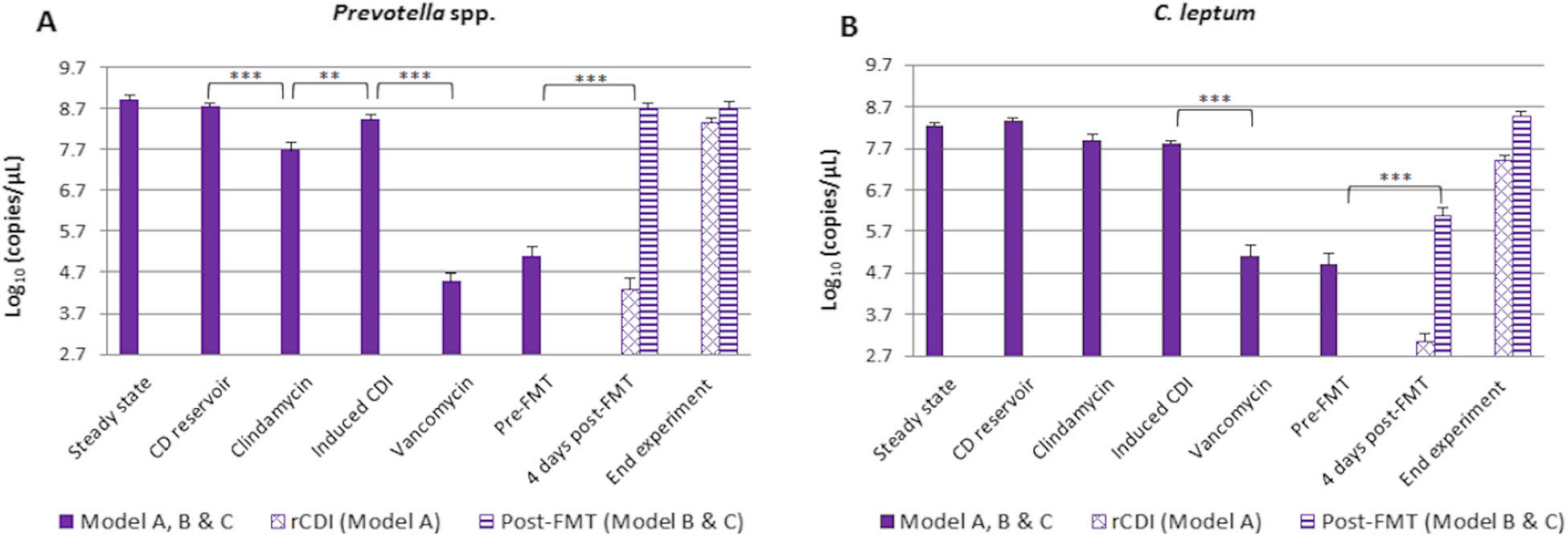
Gut microbiota populations of **a**) *Prevotella* spp., and (**b**) *C. leptum* in vessel 3 of model A, B and C, investigated by qPCR only. CD, *C. difficile*; rCDI, recurrent CDI; FMT, faecal microbiota transplantation. Asterisks represent significant variations between time points: *correspond to *p* < 0.05, **correspond to *p* < 0.005, and ***correspond to *p* < 0.0005

*Prevotella* spp. and *C. leptum* are important bacterial populations in the human microbiome [19], however; these groups could only be monitored by qPCR due to lack of selective agars available (Fig. 3). *Prevotella* spp. and *C. leptum* did not significantly change following the addition of *C. difficile* spores. Clindamycin caused a ~ 1 log_10_ copies/μL (*p* < 0.0005) decline in *Prevotella* spp., followed by recovery during CDI phase (*p* < 0.005) (Fig. 3a). Vancomycin treatment also caused a decline of ~ 4 log_10_ copies/μL (*p* < 0.0005). In model A, *Prevotella* spp. recovered by the end of the experiment. In models B and C, the FMT led to a ~ 4 log_10_ copies/μL (*p* < 0.0005) recovery within 4 days. Although a decline in *C. leptum* was observed during clindamycin instillation, this was not significant (Fig. 3b). Vancomycin dosing caused a decline of ~ 3 log_10_ copies/μL (*p* < 0.0005). Full recovery of this population was observed at the end of the experiment for all models, but in model B and C, population level increased earlier, showing a ~ 1 log_10_ copies/μL (*p* < 0.0005) recovery just 4 days post-FMT.

### Individually monitoring of selected populations

**Fig. 4:**
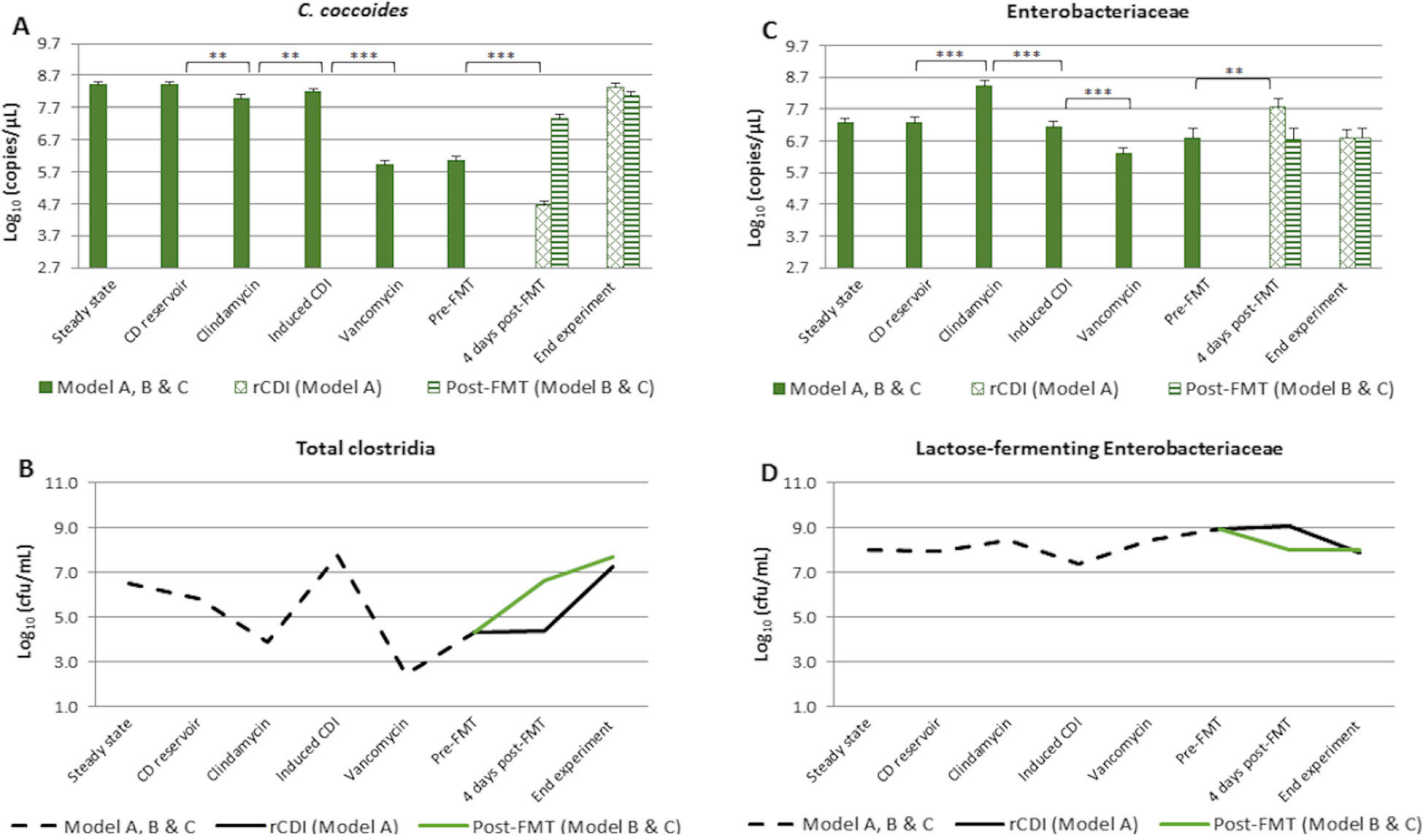
Levels of bacterial populations in vessel 3 of model A, B and C. (**a**) qPCR results for *C. coccoides* group in log_10_ copies/μL, (**b**) culture results in log_10_ cfu/mL for total clostridia, (**c**) qPCR results for Enterobacteriaceae in log_10_ copies/μL, (**d**) culture results in log_10_ cfu/mL for lactose-fermenting Enterobacteriaceae. CD, *C. difficile*; rCDI, recurrent CDI; FMT, faecal microbiota transplantation. Asterisks represent significant variations by qPCR between time points: *correspond to *p* < 0.05, **correspond to *p* < 0.005, and ***correspond to *p* < 0.0005

In this study, the qPCR results for *C. coccoides* were correlated with the culture data for *Clostridium* spp.. Among the primer pairs used in this study, the *C. coccoides* primers showed the higher specificity for the *Clostridium* species most commonly isolated from the gut model, being able to amplify 9 out of the 13 species investigated (Table 1). In addition to *Clostridium* species [17, 24], *C. coccoides* group includes species from several other genus. Due to its complexity, the populations that constitute the *C. coccoides* group were monitored by qPCR (Fig. 4a). Declines in *C. coccoides*, of ~ 1 log_10_ copies/μL (*p* < 0.005) and ~ 2 log_10_ copies/μL (*p* < 0.0005), were observed as a result of clindamycin and vancomycin dosing, respectively. In model B and C, this population recovered ~ 1.5 log_10_ copies/μL (*p* < 0.0005) post-FMT. Selective culture allowed the monitoring of several commensal clostridia species of the human microbiota (Fig. 4b). In this study, selective culture showed declines in total clostridia counts following clindamycin (*~* 2 log_10_ cfu/mL) and vancomycin (*~* 5 log_10_ cfu/mL). In model A, clostridia populations recovered within 4 weeks, whereas in model B and C a *~* 2 log_10_ cfu/mL increase was observed 4 days post-FMT. Clindamycin instillation caused an increase in both Enterobacteriaceae populations monitored by qPCR (~ 1 log_10_ copies/μL, *p* < 0.0005) (Fig. 4c) and lactose-fermenting Enterobacteriaceae monitored by selective culture (*~* 1 log_10_ cfu/mL) (Fig. 4d). qPCR analysis showed a decline of ~ 1 log_10_ copies/μL in Enterobacteriaceae following vancomycin instillation (*p* < 0.0005); however, data of direct culture showed that lactose-fermenting Enterobacteriaceae populations increased *~* 1 log_10_ cfu/mL during this stage. According to qPCR, the FMT did not significantly affect Enterobacteriaceae populations. However, culture data showed a decline of *~* 1 log_10_ cfu/mL in lactose-fermenting populations in model B and C, 4 days post-treatment.

**Table 1:** Amplification of *Clostridium* species commonly isolated in gut model samples using the *Clostridium* specific primers

Strain	Primers		
	Sg-clept-F and Sg-clept-R	Eub338F and Erec482R	Cdiff-F and Cdiff-R
*Clostridium difficile*	–	+	+
*Clostridium hathewayi*	–	+	–
*Clostridium celerecrescens/ Clostridium sphenoides*^a^	–	+	–
*Clostridium sporogenes*	–	–	–
*Clostridium paraputrificum*	–	+	–
*Clostridium tertium*	–	+	–
*Clostridium butyricum*	–	–	–
*Clostridium clostridioforme*	–	+	–
*Clostridium symbiosum*	–	+	–
*Clostridium bolteae*	–	+	–
*Clostridium innocuum*	–	–	–
*Clostridium scindens*	–	+	–
*Escherichia coli*^*b*^	–	–	–

^a^Strains sharing 98% of 16S rRNA gene homology [25], Maldi-ToF identification did not provide reliable distinction between species

^b^Included in each assay as negative control

### Correlation between bacterial culture and qPCR assays

A Pearson correlation coefficient was used to determine the relationship between bacterial populations enumerated by qPCR measurements and culture-based methods (Table 2). The higher correlation between methods was observed for bacterial genus/species targeted by both methods. This was particularly seen in *Bacteroides* spp., as the bacterial levels detected throughout the study showed a correlation of 98% between methods, suggesting these assays target the same *Bacteroides* populations within the gut models. Good correlations were also observed for *Lactobacillus* spp., *Enterococcus* spp. and *Bifidobacterium* spp., corresponding to 94, 85 and 73%, respectively. The linear relationship between methods for all these populations was > 0.85. As the models are maintained in anaerobic conditions, for the correlation analysis, the qPCR data targeting total Eubacteria was compared the counts of total bacteria cultured in anaerobic conditions, with an association of 86% between assays. Unsurprisingly, given the slightly different targets of qPCR and culture enumeration, the lower correlation values were observed for Enterobacteriaceae (62%) and clostridia (63%), with both assays showing a linear relationship of 0.79. These correlation values were highly affected by the results observed in each assay during antibiotic instillation. The correlation analysis for Enterobacteriaceae showed the most variation during vancomycin dosing. Similarly, *Clostridium* spp. direct enumeration and *C. coccoides* qPCR assay showed the most variation during clindamycin dosing, which declined the linear correlation between assays from 0.91 to the reported 0.79.

**Table 2:** Pearson correlation coefficient analysis between bacterial enumeration and qPCR in the pre FMT period

Bacterial population		R value	R^2^ value
Culture assay	16S target by qPCR		
Total bacteria (cultured anaerobically)	Total bacteria (Eubacteria)	0.93	0.86
Lactose-fermenting Enterobacteriaceae	Enterobacteriaceae	0.79	0.62
*Enterococcus* spp.	*Enterococcus* spp.	0.92	0.85
*Bacteroides* spp.	*Bacteroides* spp.	0.99	0.98
*Bifidobacterium* spp.	*Bifidobacterium* spp.	0.86	0.73
*Lactobacillus* spp.	*Lactobacillus* spp.	0.97	0.94
*Clostridium* spp.	*C. coccoides*	0.79	0.63

## Discussion

Gut microbiota variations and microbiota reconstitution were monitored in three chemostat *in vitro* models of the human colon using two methods of quantitative bacterial enumeration: culture-based assay and qPCR. Models A, B and C underwent clindamycin induced CDI followed by treatment with vancomycin. In model A, CDI was treated with vancomycin only, whereas in models B and C vancomycin treatment was followed by an FMT. Post-treatment, bacterial populations were allowed four weeks to recover.

Similar to previous *in vitro*Escherichia coli cells gut model studies [10–13] antibiotic therapy with clindamycin and vancomycin caused a decline in several of the bacterial populations monitored, particularly *Bifidobacterium* spp., *Bacteroides* spp., clostridia, *Enterococcus* spp., *Lactobacillus* spp., and *C. difficile*. Disruption of the gut microbiota was exacerbated following vancomycin dosing, with these declines being observed by both bacterial culture and qPCR.

A good correlation (> 85%) between qPCR and direct culture was observed for most populations investigated. The lowest correlation value, 62%, was observed between the qPCR assay for Enterobacteriaceae and selective culture for lactose-fermenting Enterobacteriaceae populations, partially due to the different trends observed between assays during vancomycin dosing. *Escherichia* spp., *Klebsiella* spp., *Citrobacter* spp., and *Enterobacter* spp. species are among the genera of the normal intestinal flora of humans that have the capacity to process lactose [26]. However, some genera within the Enterobacteriaceae family are non-lactose fermenters and may require other media for its selective culture [26, 27]. Among these, *Salmonella* spp., *Shigella* spp., *Serratia* spp., and *Proteus* spp. species can be part of the human gastrointestinal microbiota, and their presence was confirmed in these gut models by MALDI-TOF identification of selected colonies in non-selective nutrient agar. As the qPCR reaction targets the 16S region of the overall Enterobacteriaceae gut populations without distinguish the lactose fermenting species, this possibly explains the lower correlation observed in this case between qPCR and bacterial culture.

*Prevotella* spp., *C. coccoides* and *C. leptum* populations were investigated due to their importance for a healthy microbiome [16, 19, 20, 24]. We found that direct enumeration of *Clostridium* spp. correlated with the qPCR results for *C. coccoides* based on the specificity of this primer pair for clostridia species commonly isolated from gut model samples; however, not all *Clostridium* species were amplified. Correlation between culture of *Clostridium* spp. and the qPCR assay for *C. coccoides* mostly differed during clindamycin dosing period, when culture showed a high decline in clostridia counts that was less noticeable by qPCR. Within the *Clostridium* genus, *C. coccoides* (*Clostridium* cluster XIVa) and *C. leptum* (*Clostridium* cluster IV) are two highly predominant groups of the human gastrointestinal tract [16, 17, 19, 24]. Each of these groups is complex, with *C. cocoides* being closer in composition to the Lachnospiraceae family and *C. leptum* to the Ruminococcaceae family. *C. coccoides* includes *Butyrivibrio, Clostridium, Coprococcus, Dorea, Eubacterium, Lachnospira, Roseburia*, and *Ruminococcus* species, and *C. leptum* includes *Clostridium, Ruminococcus, Eubacterium* and *Faecalibacterium prausnitzii* species [17, 18, 24, 28]. Since several bacterial populations are represented in the *C. coccoides* group, it is likely that some species within the group were less disturbed by clindamycin instillation, which was reflected in the qPCR assay results.

In this study, *C. difficile* was investigated using two qPCR assays, one for the species 16S region and other for the housekeeping gene *gluD*. Results consistently showed *C. difficile* levels ~ 1 log_10_ copies/μL higher in the 16S gene assay compared to the *gluD* assay. Concentrations of *C. difficile* genomic DNA ranging from 12.5 ng to 0.024 ng were tested with both primer sets, showing results ~ 1.5 log_10_ copies/μL higher in the 16S assay compared to *gluD*. As *C. difficile* genome has 11 copies of the 16S rRNA operon and 1 copy of the *gluD* gene [29] this could possibly explain the apparent higher sensitivity of the 16S qPCR assay.

Overall, both quantitative analyses of gastrointestinal bacterial populations showed an earlier reconstitution of the gut microbiota in the models that received an FMT. Although both assays provide absolute quantification data, they have different limits of detection. As example, *Bifidobacterium* spp. was periodically undetectable by direct culture, whereas all commensal populations remained detected by qPCR throughout the experiment. Although the assays showed a good correlation, an improvement is likely to be observed if the limits of detection of both methodologies are taken into consideration. qPCR allowed us to monitor bacterial groups that would otherwise not be possible to investigate and offered a shorter turnaround time (less than 24 h) for results compared with culture. In addition to its high sensitivity, qPCR also allowed analysis of higher number of technical replicates adding the possibility of statistical analysis. However, by detecting DNA, we are unable to distinguish between viable and non-viable cells. Despite having a slower turnaround time, particularly for anaerobic populations that require 48 h incubation, the culture results reflect cell viability and coupled with MALDI-TOF identification, provided additional quantitative information on the most abundant bacterial species in the gut model at a given time. This allows for a better understanding of any specific pathways that may be active and their potential relevance in disease. Studies involving monitoring of multiple populations by culture assay can be challenging, however, for the phylogenetically inconsistent clostridia genus [28], the use of a single selective medium in this study allowed the monitoring of high abundant *Clostridium* species. Additionally, with bacterial culture, we were able to monitor *C. difficile* at different stages of the bacteria life cycle, by differentiate spores and vegetative cells which was not possible using qPCR.

## Conclusions

Overall, the commensal bacterial populations monitored in this study showed a quicker recovery in the models that received an FMT, compared to treatment with vancomycin only. Alongside this recovery, the models that received an FMT did not show recurrent infection, whereas model A showed *C. difficile* proliferation and toxin production, consistent with rCDI. These results show the potential of replacement therapies in the reconstitution of the normal human microbiota. Furthermore, the qPCR assays correlated well with the results of bacterial enumeration by culture, with both methods showing antibiotic-mediated depletion of the gut microbiota populations, followed by reconstitution of gastrointestinal populations within four weeks. The molecular assays can potentially provide information on gut bacterial variations in real-time, reducing the standard requirements for bacterial culture, and allowing the monitoring of bacterial groups that would otherwise not be possible. However, in some cases it may not be possible to monitor an individual genus without selective culture.

## Methods

### Chemostat model assembly and experimental design

Three triple-stage chemostat models, assembled as previously described [9, 14] were run simultaneously. Briefly, each model consists of three glass vessels displayed in a weir cascade and kept at 37 °C by a water jacket. This system simulates the nutrient availability and pH conditions of the proximal, medial and distal colon in vessel 1, 2 and 3, respectively. The models are continuously fed with a complex nutritive medium (Table 3), at the pre-established dilution rate of 15 mL/h and an anaerobic environment was maintained by sparging the system with nitrogen. All models were inoculated with a slurry of pooled human faeces [10% w/v in pre-reduced phosphate buffered saline (PBS)] from 22 healthy volunteers, > 60 years of age, and with no history of antibiotic therapy in the previous 6 months. In each model, bacterial populations were allowed to equilibrate for 4 weeks, followed by addition of two doses of 10^7^ cfu/mL *C. difficile* spores, a week apart. *C. difficile* spores of the PCR ribotype 027 strain 210 were prepared as described before [30], and added to the model. CDI was induced following clindamycin instillation (33.9 mg/L, four times daily for 7 days) (Fig. 1). *C. difficile* germination, growth and toxin production were monitored and at peak toxin production, all models were dosed with vancomycin (125 mg/L, four times daily for 7 days). Antibiotic treatments were based on previously reported faecal concentrations and prescribing recommendations for these drugs [12, 31]. Three days post vancomycin, model A was left without further intervention, whereas models B and C were treated with an FMT preparation. Up to the end of vancomycin treatment, all models followed the same experimental design, representing replicates for simulated CDI and treatment, as previously described [11–13]. Afterwards, model A proceeded as a representation of a simulated rCDI case, whereas model B and C proceeded as replicates of a simulated FMT treatment. The FMT experiment was performed in duplicate to confirm method reproducibility in the chemostat model. FMT was prepared similar to the initial slurry, by performing a 10-fold dilution with pre-reduced PBS of a faecal sample donated by a relative of one of the initial donors, aged 30, without history of antibiotic use in the previous 6 months. The FMT (50 mL) was added to the bottom of vessel 1 of models’ B and C at a 50 mL/h rate using a peristaltic pump. Microbial populations were monitored for 4 weeks post instillation in all models.

**Table 3:** Target populations and agar composition for bacterial ennumeration

Gut model growth medium Solid components (g/L): Peptone water (2.0), yeast extract (2.0), NaCl (0.1), K2HPO4 (0.04), KH2PO4 (0.04), MgSO4.7H2O (0.01), CaCl2.2H2O (0.01), NaHCO3 (2.0), haemin (0.005), cysteine HCl (0.5), bile salts (0.5), glucose (0.4), arabinogalactan (1.0), pectin (2.0), starch (3.0) Liquid components: vitamin K1 at 10 mL/L, and Tween 80 at 0.2% Medium is sterilised at 121 °C for 30 min and cooled to 50 °C. Post-autoclaving glucose (0.4 g/L), and resazurin anaerobic indicator (0.005 g/L) are added.			
Target populations	Agar	Supplements	Incubation (temp, environment)
Total anaerobes and total *Clostridium* spp.	Fastidious anaerobe agar	5% horse blood	37 °C, anerobic
*Bifidobacterium* spp.	42.5 g/L Columbia agar, and 5 g/L agar technical	0.5 g/L cysteine HCl, 5 g/L glucose	37 °C, anerobic
*Bacteroides spp.*	Bacteroides bile aesculin agar	5 mg/L haemin, 10 μL/L vitamin K, 7.5 mg/L vancomycin, 1 mg/L penicillin, 75 mg/L kanamycin and 10 mg/L colistin	37 °C, anerobic
*Lactobacillus* spp.	52.2 g/L MRS broth and 20 g/L agar technical	0.5 g/L cysteine hydrocloride, 20 mg/L vancomycin	37 °C, anerobic
Total facultative anaerobes	Nutrient agar	N/A	37 °C, aerobic
Lactose fermenting Enterobacteriaceae	MaConkey’s agar	N/A	37 °C, aerobic
*Enterococcus* spp.	*Kanamycin* aesculin *azide agar*	10 mg/L nalidixic acid, 10 mg/L aztreonam, and 20 mg/L kanamycin	37 °C, aerobic
Total spores (following alcohol shock for 1 h)	Fastidious anaerobe agar	5% horse blood	37 °C, anerobic
*C. difficile* total viable cells	Braziers CCEY agar	D-cycloserine (250 mg/L) cefoxitin (8 mg/L), 5 mg/L lysozyme, and 20 mL/L lysed horse blood	37 °C, anerobic
*C. difficile* spores	Braziers CCEY agar	5 mg/L lysozyme, and 2% lysed horse blood	37 °C, anerobic

### Bacterial culture and cytotoxin assay

Models were sampled for culture profiling of key intestinal microbiota populations using selective and non-selective agars described in Table 3. Populations of total bacteria, *Clostridium* spp. lactose-fermenting Enterobacteriaceae, *Enterococcus* spp., *Bacteroides* spp., *Bifidobacterium* spp., *Lactobacillus* spp., *C. difficile* total viable cells and *C. difficile* spores were monitored at the time points illustrated in Fig. 1. All bacterial populations and *C. difficile* spores were cultured as previously described [9]. Briefly, *C. difficile* spores were isolated by treating 0.5 mL of gut model fluid with 0.5 mL of 96% ethanol. The samples were incubated at room temperature for 1 h, serially diluted to 10^− 3^ in peptone water, and 20 μL of each sample dilution was plated in triplicate onto supplemented Braziers CCEY agar (Table 3). Plates were incubated anaerobically for 48 h and distinctive colonies were enumerated. Identification of bacterial isolates were confirmed by Matrix-Assisted Laser Desorption Ionization-Time of Flight (MALDI-TOF). Vessel 3 was particularly investigated due to its high microbial content [32] and because it represents the area of the colon more physiologically relevant for CDI [33]. The limit of detection for culture assay was ~ 1.22 log_10_ cfu/mL.

The *C. difficile* toxin levels were measured by cell cytotoxicity assay, as previously described [9]. Briefly, model fluid was centrifuged, filtered, and serially diluted on a 10-fold series. Each dilution was inoculated onto a confluent monolayer of Vero cells and incubated for 48 h at 37 °C at 5% CO_2_. Samples were considered positive for *C. difficile* toxin when > 70% cell rounding was observed. Results are expressed as RUs, as follows: positive result on a 1:10 dilution = 1 RU, 1:100 = 2 RU, etc.

### DNA extraction of gut model samples

For molecular analysis, samples were taken from vessel 3 of each model as outlined in Fig. 1. For each time point, DNA extraction was performed in triplicate using the FastDNA Spin kit for Soil (Mpbio) following manufacturer’ instructions except that sample homogenisation was performed using Precellys 24 (Bertin Instruments) at 6500 rpm for 40 s. DNA concentration was measured using a Nanodrop 2000c and each sample was normalised to 5 ng/μL.

### Preparation of control curves using plasmid standard DNA

For the analysis of *Enterococcus* spp., *Bifidobacterium* spp., *Lactobacillus* spp., and *C. difficile*, plasmids containing 16S gene inserts specific for these populations were prepared as described before [23]. Group-specific PCR reactions were performed on appropriate bacterial strains using the primers described in Table 4. The products were cloned using the TOPO TA cloning kit (Thermo Fisher Scientific) and inserts were verified by Sanger sequencing capillary electrophoresis (Applied Biosystems). Bacterial group specificity of the insert in transformed colonies was confirmed by blastn search and plasmid DNA was isolated using QIAprep spin miniprep kit (Qiagen). Plasmids containing 16S gene inserts for total bacteria, Enterobacteriaceae, *Bacteroides* spp., *Prevotella* spp., *Clostridium coccoides* and *Clostridium leptum* were provided by Dr. Cheleste M. Thorpe (Tufts Medical Center, USA) [23]. Plasmids were re-suspended, propagation was performed using TOP10 chemically competent *Escherichia coli* cells (Thermo Fisher Scientific) according to manufacturers’ instructions, and plasmid DNA was obtained as above. The molecular weight of each plasmid containing 16S gene inserts was determined using a web calculator (http://www.encorbio.com/protocols/Nuc-MW.htm). Purified plasmid concentrations were determined using Qubit 2.0 Fluorometer and 10-fold dilution series ranging from 5 × 10^9^ copies/μL to 500 copies/μL were prepared.

**Table 4:** Sequence of primers used for each bacterial group

Target	Primer	Sequence (5′-3′)	Product size (bp)	Annealing (time, temp)	Reference
*Bacteroides* spp.	Bac303F	GAAGGTCCCCCACATTG	419	45 s, 54 °C	[34]
	Bac708R	CAATCGGAGTTCTTCGTG			
Enterobacteriaceae	Eco1457F	CATTGACGTTACCCGCAGAAGAAGC	190	45 s, 60 °C	[21]
	Eco1652	CTCTACGAGACTCAAGCTTGC			
*C. leptum* group	Sg-clept-F	GCACAAGCAGTGGAGT	241	45 s, 54 °C	[19]
	Sg-clept-R	CTTCCTCCGTTTGTCAA			
*C. coccoides* group	Eub338F	ACTCCTACGGGAGGCAGC	139	30s, 56 °C	[20]
	Erec482R	GCTTCTTAGTCANGTACCG			
*Prevotella spp.*	CFB286F	GTAGGGGTTCTGAGAGGA	446	30s, 56 °C	[20]
	CFB719R	AGCTGCCTTCGCAATCGG			
Eubacteria	8F	AGTTTGATCCTGGCTCAG	417	45 s, 54 °C	[35]
	515R	GNATTACCGCGGCNGCTG			
Probe	338P	FAM GCTGCCTCCCGTAGGAGT BHQ1			
*Bifidobacterium spp.*	Bif551F	CGCGTCNGGTGTGAAAG	244	20s, 55 °C	[36]
	Bif794R	CCCCACATCCAGCATCCA			
*Lactobacillus spp.*	Lacto-F	GAGGCAGCAGTAGGGAATCTTC	126	45 s, 60 °C	[36]
	Lacto-R	GGCCAGTTACTACCTCTATCCTTCTTC			
*Enterococcus spp.*	Enter-F	CCCTTATTGTTAGTTGCCATCATT	144	20s, 56 °C	[22]
	Enter-R	ACTCGTTGTACTTCCCATTGT			
*C. difficile* (16S gene)	Cdiff-F	CAAGTTGAGCGATTTACTTCGGTAA	177	20s, 59 °C	[37]
	Cdiff-R	CTAATCAGACGCGGGTCCAT			
*C. difficile* (GluD gene)	GluD-F	ATGCAGTAGGGCCAACAAAA	135	20s, 55 °C	[38]
	GluD-R	TTCCACCTTTACCTCCACCA			

### qPCR assays and data analysis

The DNA levels of each bacterial genus/species were assessed using primers previously validated for qPCR (Table 4). Reactions containing final concentration of SYBR Green 1x Master Mix (Qiagen), 0.3 μM primers and 12.5 ng of DNA template were prepared to a final volume of 10 μL. A FAM-tagged probe at 0.25 μM was added to the Eubacteria reaction mix [23]. Reactions were analysed in a 7500 Real-Time PCR System (Thermo Fisher). DNA extraction replicates were run in duplicate for all bacterial populations investigated. Plasmid DNA standard curves were included on each qPCR plate in triplicate and used to convert threshold cycle values to copies per μL of template. The same concentration (12.5 ng) of DNA template and plasmid DNA standard was used in each reaction. Standard curve R square values ranged from 0.990 ± 0.009. Limit of detection was established at 500 copies. The change in bacterial levels were calculated based on logarithms of 16S rRNA gene copy numbers to achieve normal distribution. GraphPad Prism was used for analysis of log transformed data. Statistical significance between time-points was assessed using a two-sided Wilcoxon Signed Rank test with a 95% confidence interval. *P* ≤ 0.05 was considered statistically significant.

### Study of *Clostridium* species

*C. difficile* was investigated using two qPCR assays, one targeting the housekeeping gene *gluD*, and another targeting the 16S gene. Specificity of the primer sets was investigated by standard PCR. DNA template of *Clostridium* species isolated from gut model samples and identified by MALDI-TOF were amplified using the primers for *C. coccoides* group, *C. leptum* group and *C. difficile* 16S gene (Table 4) to determine primer specificity (Table 1).

## Supplementary information

**Additional file 1:** **Figure S1.** Mean gut microbiota populations of *C. difficile* based on the housekeeping gene *gluD* in vessel 3 of model A, B and C at the different stages of the experiment. Bar graphs represent the levels in log_10_ copies/μL measured by qPCR. CD, *C. difficile*; rCDI, recurrent CDI; FMT, faecal microbiota transplantation. Asterisks represent significant variations by qPCR between time points: *correspond to *p* < 0.05, and ***correspond to *p* < 0.0005. (TIF 619 Kb)

## Data Availability

The data supporting the conclusions of this article are presented in this published article, and in the additional file 1: Figure S1. Full datasets are available from the corresponding author on reasonable request.

## Abbreviations

CCEY: Cycloserine, cefoxitin egg yolk
CDI: *Clostridioides difficile* infection
cfu: Colony forming unit
FMT: Faecal microbiota transplantation
MALDI-TOF: Matrix-Assisted Laser Desorption Ionization-Time of Flight
N/A: Not applicable
PBS: Phosphate buffered saline
qPCR: Quantitative real-time PCR
rCDI: Recurrent CDI
rpm: Rotations per minute
rRNA: Ribosomal ribonucleic acid
RUs: Relative units
