# Supplementary figures and images for: Method comparison for the direct enumeration of bacterial species using a chemostat model of the human colon
Source: BMC Microbiol. 2020 Jan 2;20:2. doi: 10.1186/s12866-019-1669-2 (PMC6941270; doi:10.1186/s12866-019-1669-2)

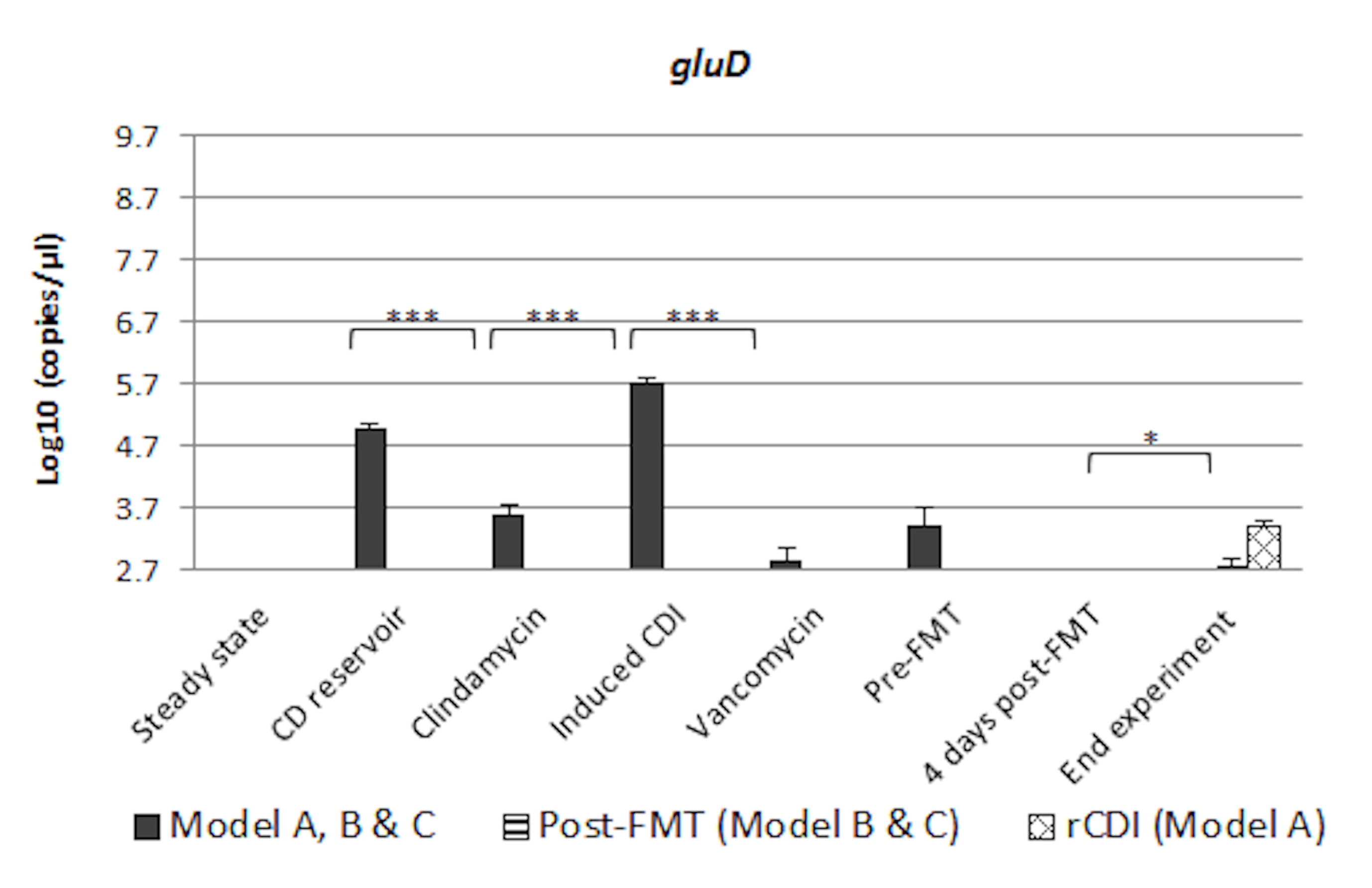

Supplement: Supplementary file 1 — Additional file 1: Figure S1. Mean gut microbiota populations of C. difficile based on the housekeeping gene gluD in vessel 3 of model A, B and C at the different stages of the experiment. Bar graphs represent the levels in log10 copies/μL measured by qPCR. CD, C. difficile; rCDI, recurrent CDI; FMT, faecal microbiota transplantation. Asterisks represent significant variations by qPCR between time points: *correspond to p < 0.05, and ***correspond to p < 0.0005. (TIF 619 Kb) [file 12866_2019_1669_MOESM1_ESM.tif]
